# Energy and nutrient content of school lunches provided for children attending school-based nurseries: a cross-sectional study

**DOI:** 10.1017/S1368980023002331

**Published:** 2023-12

**Authors:** Claire J Wall, Jo Pearce

**Affiliations:** Food & Nutrition Subject Group, Sheffield Business School, Sheffield Hallam University, Howard Street, Sheffield, S1 1WB, UK

**Keywords:** School lunch, nursery, early years, children, nutrient analysis

## Abstract

**Objective:**

To nutritionally analyse lunches provided for 3–4-year-old children attending school nurseries. Energy and nutrient content are compared with nutrient frameworks underpinning voluntary guidelines for early years settings (EYS) and mandatory standards for infant schools (4–7-year-olds).

**Design:**

A cross-sectional study, recording all main meals, vegetarian meals, jacket potato options, sandwich options and all desserts and accompaniments provided over 5 consecutive days in each school. Two portions of each meal were collected each day and weighed. Recipe and portion weight data were entered into nutrient analysis software.

**Setting:**

School nurseries where lunch was provided by the school.

**Subjects:**

Nine schools, providing a total of 161 meals.

**Results:**

Lunches contained more energy (1881 kJ/450 kcal), fat (15·5 g), free sugars (10·5 g) and Na (424 mg) than suggested by the nutrient framework for EYS. Carbohydrate (60·6 g), protein (16·8 g), fibre (6·7 g), Fe (2·4 mg), Zn (2·0 mg), Ca (202 mg), vitamin A (304 µg) and vitamin C (19 mg) also exceeded minimum recommendations. Compared with a revised nutrient framework for infant schools, energy was within range, whilst saturated fat, free sugars and Na were above maximum recommendations for this age group, and Zn was below. Sandwich meals were lower in vitamin C (*P* < 0·001–*P* = 0·05) and Fe (*P* = 0·012–*P* = 0·017) and higher in Na (*P* < 0·001–*P* = 0·003) and Ca (*P* < 0·001–*P* = 0·05).

**Conclusion:**

Lunches provided for children attending school nurseries are more in line with the framework for 4–7-year-olds. Free sugars, saturated fat and Na are areas of concern consistent with previous studies. Protein is three times more than recommended. Large portions of cakes and biscuits contribute to excess energy provision.

A balanced diet during children’s early years is vital to meet nutritional requirements, support appropriate growth and development and to promote healthy eating habits^(1–3)^. However, diets of young children in the UK do not meet all recommendations in place for this age group. Intakes of free sugars are approximately double maximum recommendations and mean dietary fibre intakes are low^(4)^. Although dietary reference values (DRV) for fat and saturated fat do not fully apply until 5 years of age, intakes of saturated fat in young children are considerably higher than the recommended maximum 10 % of dietary energy,^(4)^ and mean protein intakes are more than twice the reference nutrient intake, which may be associated with increased BMI in later childhood^(4,5)^. In addition, intakes of micronutrients including Fe, Zn and vitamin A may be inadequate amongst children from lower socio-economic or minority ethnic groups^(4)^. Intakes of salt in young children are also above maximum recommended levels^(4,6)^.

All 3- and 4-year-old children in England are entitled to funded early education to support child development and school readiness, with children of working parents eligible for an additional entitlement^(7)^. There were an estimated 1·54 million registered early years places in England in 2022, with approximately 20 % being school-based nursery places^(8)^. Over 90 % of 3- and 4-year-old children are registered to receive this entitlement,^(7)^ and further expansion of funded childcare is planned over the next 2 years^(9)^, providing an important opportunity for early years settings (EYS) to support healthy dietary habits for young children^(10)^ and to reduce health inequalities^(11)^.

Adherence to food or nutrient-based standards in EYS in England is not mandatory, but food provided is required to be ‘healthy, balanced and nutritious’^(12)^. Voluntary government-funded food and drink guidelines were published in 2012 to support settings to interpret this requirement^(13)^ and were updated in 2017 following revisions to UK dietary recommendations for energy, free sugars and fibre^(14)^. These guidelines are food based but are underpinned by a nutrient framework based on DRV for children aged 1–4 years^(15)^. Other national and local guidance on food provision and portion sizes is also available, but often targeted at parents rather than childcare settings specifically^(16)^. Conversely, most schools in England are required to follow mandatory food-based standards for school lunches, with the current standards introduced in 2015^(17,18)^. These standards are also underpinned by a nutrient framework based on the nutritional requirements of children aged 4–11 years. The nutrient framework is consistent with previous nutrient-based standards that were in place in England from 2008 to 2014, which also included nutrient-based standards for standalone infant schools (catering for key stage one (KS1) children aged 4–7 years).^(19,20)^. Unlike guidance for EYS, these standards have not been updated to reflect changes to dietary recommendations (e.g. for free sugars). Lunches provided to children attending school nurseries are not required to meet the food-based standards in place for schools and instead are simply required to include food from each of the main food groups^(17)^. The food and drink guidelines for EYS were designed to be used by all setting types, including school nurseries. Schools may therefore be attempting to adhere to both school food standards and guidelines for EYS using one menu, and the extent to which the guidelines for EYS are met for the youngest children is not clear.

Previous research into food provision and practices in EYS was conducted either before the introduction of the voluntary food and drink guidelines or before the guidelines were updated in 2017^(21–24)^. Studies evaluating the energy and nutrient content of lunches reported concerns that meals provided were low in energy, carbohydrate, Fe and Zn and high in Na^(23,24)^. Meanwhile, a nationally representative evaluation of the nutrient content of primary school lunches (completed before the introduction of the current food-based standards) revealed meals were higher than recommended in energy, sugars and Na^(25)^. More recently, evaluation of school lunches provided across the UK between 2008 and 2017 revealed that for KS1 children, intakes of Ca and Fe were the least likely of the nutrients analysed to meet recommendations^(26)^. There is a lack of evidence on the current energy and nutrient content of lunches provided within EYS and schools and how these compare with relevant standards and guidance, and no studies focusing specifically on the nutrient content of lunches provided to children attending school nurseries have been identified. This study aimed to nutritionally analyse lunches provided for 3–4-year-old children attending school nurseries and compare the energy and nutrient content of these to the nutrient frameworks underpinning voluntary guidelines for EYS and standards applying to infant schools to evaluate whether provision was in line with DRV for children of this age.

## Methods

### School recruitment

Ethical approval was granted by Sheffield Hallam University research ethics review system (ID: ER38429936). A detailed description of the school selection and data collection methods was published previously^(27)^. In brief, infant and primary schools with nursery provision in Sheffield and the surrounding areas were identified using the government’s school database^(28)^ with the aim of recruiting ten schools to participate. Schools were only eligible to participate if school lunches were available to children attending the nursery, and a maximum of two schools using any single catering provider were recruited.

Each recruited school was visited for five consecutive school days, between February and July 2022. On each day of data collection, school kitchen staff provided two portions of each main meal (including any starchy and/or vegetable accompaniments), two portions of each vegetarian meal (including any starchy and/or vegetable accompaniments), two portions of each dessert and one portion each of any jacket potato and sandwich options available to children within the nursery. Kitchen staff were asked to serve food as they would for nursery children, on their normal plates or trays. Each meal was separated into individual components (e.g. meat, potatoes and vegetables), and composite dishes were further disaggregated where possible into separate ingredients (e.g. the chicken separated from sauce in a curry). Meal components and ingredients were then weighed using kitchen scales (Salter), and mean portion weights were calculated for each food item and recorded separately for each meal type (e.g. carrots served as part of the main meal would have a different weight from carrots served as part of the vegetarian meal).

### Menu and nutrient analysis

Main meals (including accompaniments and desserts) provided across the week of collection in each school were evaluated against the food-based standards included within the current school food standards^(18)^ and the voluntary food and drink guidelines for EYS in England relating to lunch provision^(14)^ to determine the extent to which actual lunch provision met current standards and guidelines. Adherence to standards and guidelines which apply over a period of more than 1 week (e.g. the requirement to provide oily fish at least once every 3 weeks) or that are related to provision outside of the meal (e.g. whether condiments were available for children to use and whether drinking water was available in the dining room) was not evaluated.

Menu and recipe information was collected from each school. Recipes contained details of the ingredients, ingredient weights, products, product codes, cooking methods and cooking time. Recipes were entered into Nutritics^(29)^ by two researchers (both Registered Nutritionists, experienced in the use of nutrient analysis software and evaluation of school food provision), who resolved queries regarding missing or unclear information by consensus. Ingredients were entered as raw ingredients wherever possible, with cooking method applied to each ingredient, to adjust for nutrient losses during cooking. An overall weight change factor was also applied for each recipe, using standard options in Nutritics^(29)^. Wherever product information, product names or product codes were provided, new foods were inputted per 100 g using data from the distributor, retailer or manufacturers’ websites. Where a food (entered as part of a recipe) had missing micronutrient information, the nearest match was chosen to avoid underestimation of micronutrient content. To avoid overestimation of Na or free sugars, lower salt and sugar-free options were chosen where it was unclear whether standard or alternative options had been provided by the school (e.g. where just ‘jelly’ was stated, sugar-free jelly was assumed). Standard food codes were chosen from Nutritics (based on data from McCance & Widdowson) for foods such as ‘boiled carrots’ or ‘raw cucumber’^(29,30)^. Ten percent of recipe analysis was checked to ensure consistency and reliability.

Menus were created in Nutritics for each meal type (main meal, vegetarian main meal, jacket potato option and sandwich option) provided across the week in each school, and the energy and nutrient content of an average daily lunch provided for each meal type in each school was calculated. Each food item or recipe was entered along with its exact portion weight, using the portion weight data collected for that meal. Caterers taking part in the study did not always provide the stated vegetable/salad accompaniments alongside sandwich or jacket potato options. Where this was the case and it was indicated on the menu that there should have been accompaniments, the mean portion weights of these items provided with main and vegetarian meals were included in the analysis.

Schools usually had more than one dessert option per day, with children able to choose from a dessert listed on the menu, or an alternative, typically fruit and/or yoghurt. The analysis outlined in Tables 1–4 is based on equal weighting of all available dessert options, for example, where a cake, a yoghurt and a piece of fruit were available, a third of a portion of cake, a third of a portion of yoghurt and a third of a portion of fruit were used in the main analysis. Given that the main dessert option (typically a cake or biscuit) would likely be more popular and more frequently chosen than the alternatives, an additional analysis of the main meals, using just the main dessert option was later completed, to investigate the impact this had on provision of key nutrients.

**Table 1:** Mean energy and nutrient content of school lunches served for 3–4-year-old children (all schools, *n* 9). Compared with the nutrient framework for lunches in early years settings

Nutrient	Nutrient framework for lunches in early years settings^(14)^	Mean energy/nutrient content (all schools, *n* 9)	Meets (✓)/does not meet (✗) nutrient framework	Number of schools meeting nutrient framework (*n* 9)	Range of mean energy/nutrient content across all schools (*n* 9)
Energy (kJ)	∼ 1542†	1881	✗	0	1371–2366
kcal	∼ 369	450	✗	0	328–566
Fat (g)	∼ 14·4†	15·5	✗	2	9·0–21·0
Saturated fat (g)*	N/A	5·8	–	–	3·1–8·3
Total carbohydrate (g)	∼ 49·2†	60·6	✗	1	44·8–75·2
Dietary fibre (g)	≥ 4·5	6·7	✓	9	5·2–8·8
Free sugars (g)	≤ 4·9	10·5	✗	0	5·6–15·6
Protein (g)	≥ 5·1	16·8	✓	9	13·6–20·0
Fe (mg)	≥ 2·3	2·4	✓	7	1·9–2·8
Zn (mg)	≥ 1·7	2·0	✓	8	1·6–2·7
Ca (mg)	≥ 120	202	✓	9	170–240
Vitamin A (µg)	≥ 136	304	✓	8	111–486
Vitamin C (mg)	≥ 9	19	✓	9	10–24
Na (mg)	≤ 300	424	✗	0	338–538
% of energy from	DRV				
Carbohydrate	∼ 50	51	–	–	47–55
Free sugars	≤ 5	9	–	–	6–13
Fat	∼ 35	31	–	–	24–33
Saturated fat	≤ 10	11	–	–	9–13
Protein	N/A	15	–	–	14–18

kJ, kilojoule; Kcal, kilocalorie; g, gram; N/A, not applicable; mg, milligram; µg, microgram; DRV, dietary reference value.

* Saturated fat is not included in the nutrient framework underpinning voluntary food and drink guidelines for early years settings but has been included in the analysis for completeness as recommendations apply from 5 years and saturated fat is included in the nutrient framework underpinning school food standards.

† Quantities of energy, fat and total carbohydrate are stated in the nutrient framework as ‘approximately’ 369 kcal, 14·4 g and 49·2 g, respectively. Quantities within 5 % of the stated amount have been classed as meeting the nutrient framework (351–387 kcal, 13·7–15·1 g fat and 46·7–51·7 g carbohydrate).

**Table 2:** Mean energy and nutrient content of school lunches served for 3–4-year-old children by school. Compared with the nutrient framework for lunches in early years settings

Nutrient	Nutrient framework for lunches in early years settings^(14)^	School 1	School 2	School 3	School 4	School 5	School 6	School 7	School 8	School 9
Energy (kJ)	∼ 1542	2366	1701	1379	1371	2374	1789	1848	1986	2157
kcal	∼ 369	566	407	330	328	568	428	442	475	516
Fat (g)	∼ 14·4	20·6	14·2	9·0	10·4	21·0	15·3	14·6	15·6	19·1
Saturated fat (g)	N/A	7·9	4·9	3·1	3·8	8·3	6·2	4·7	5·9	7·1
Total carbohydrate (g)	∼ 49·2	74·8	54·8	48·5	44·8	75·2	53·3	61·0	66·8	67·8
Dietary fibre (g)	≥ 4·5	8·8	5·4	5·9	5·2	7·1	7·8	7·6	7·6	6·1
Free sugars (g)	≤ 4·9	10·8	14·6	5·6	7·4	15·6	9·1	9·4	11·5	10·7
Protein (g)	≥ 5·1	20·0	14·8	13·9	13·6	19·2	19·0	16·7	16·7	18·1
Fe (mg)	≥ 2·3	2·7	2·2	2·2	1·9	2·8	2·4	2·5	2·4	2·4
Zn (mg)	≥ 1·7	2·5	1·6	1·7	1·8	2·2	2·7	1·9	1·8	2·1
Ca (mg)	≥ 120	238	170	173	185	240	221	200	203	203
Vitamin A (µg)	≥ 136	472	199	111	272	219	393	486	401	277
Vitamin C (mg)	≥ 9	21	10	14	16	27	21	22	20	24
Na (mg)	≤ 300	505	347	460	338	538	400	413	387	441

kJ, kilojoule; Kcal, kilocalorie; g, gram; N/A, not applicable; mg, milligram; µg, microgram.

**Table 3:** Energy and nutrient content of school lunches served for 3–4-year-old children (all schools, *n* 9). Comparison between different menu options

Nutrient	Nutrient framework for lunches in early years settings^(14)^	Energy/nutrient content – main menu option (*n* 45)		Energy/nutrient content – vegetarian option (*n* 45)		Energy/nutrient content – jacket potato option (*n* 39)		Energy/nutrient content – sandwich option (*n* 32)	
		Mean	Median	Mean	Median	Mean	Median	Mean	Median
Energy (kJ)	∼ 1542	1864	1835	1902	1797	1818	1818	1935	1772
kcal	∼ 369	446	439	455	430	435	435	463	424
Fat (g)	∼ 14·4	15·5	15·5	16·0	14·5	13·2	13·4	17·4	15·6
Saturated fat (g)	N/A	5·1	4·6	5.5	4·4	6·0	5·7	6·8	7·0
Total carbohydrate (g)	∼ 49·2	58·6	58·9	62·7	62·6	61·2	61·2	60·1	57·3
Dietary fibre (g)	≥ 4·5	6·2	6·0	7·6	7·5	8·2	7·7	4·6	4·6
Free sugars (g)	≤ 4·9	10·4	10·3	10·2	8·9	10·9	10·4	10·7	8·8
Protein (g)	≥ 5·1	18·2	17·9	15·0	13·8	17·5	17·7	16·3	15·5
Fe (mg)	≥ 2·3	2·2	2·1	2·7	2·4	2·5	2·5	2·0	2·0
Zn (mg)	≥ 1·7	2·0	1·9	2·2	2·0	2·1	2·0	1·8	1·8
Ca (mg)	≥ 120	153	146	198	190	214	187	264	271
Vitamin A (µg)	≥ 136	280	151	319	178	304	221	315	202
Vitamin C (mg)	≥ 9	21	17	21	17	21	18	12	9
Na (mg)	≤ 300	407	344	417	368	364	341	531	470

kJ, kilojoule; Kcal, kilocalorie; g, gram; N/A, not applicable; mg, milligram; µg, microgram.

**Table 4:** Energy and nutrient content of average school lunches served for 3–4-year-old children (all schools, *n* 9). Compared with the nutrient framework for lunches for KS1 pupils and revised KS1 nutrient framework to reflect current DRV

Nutrient	Nutrient framework for school lunches – KS1 pupils^(20)^		Nutrient framework for school lunches – KS1 pupils. Revised to reflect current DRV		Mean energy/nutrient content (all schools, *n* 9)	Meets (✓)/Does not meet (✗) revised nutrient framework
Energy (kJ)	∼ 2044	1940–2144	∼ 1793	1705–1881*	1881	✓
Energy (kcal)	∼ 489	464–513	∼ 429	408–450*	450	✓
Fat (g)	≤ 19·0		≤ 16·7†		15·5	✓
Saturated fat (g)	≤ 6·0		≤ 4·8‡		5·8	✗
Total carbohydrate (g)	≥ 65·2		≥ 57·2§		60·6	✓
Dietary fibre (g)	≥ 3·9		≥ 4·5^||^		6·7	✓
Non-milk extrinsic sugars (g)	≤ 14·3		N/A		N/A	N/A
Free sugars (g)	N/A		≤ 5·7**		10·5	✗
Protein (g)	≥ 5·9		≥ 5·9		16·8	✓
Fe (mg)	≥ 2·1		≥ 2·1		2·4	✓
Zn (mg)	≥ 2·3		≥ 2·3		2·0	✗
Ca (mg)	≥ 158		≥ 158		202	✓
Vitamin A (µg)	≥ 140		≥ 140		304	✓
Vitamin C (mg)	≥ 10·5		≥ 10·5		19	✓
Folate (µg)	≥ 35		≥ 35		48	✓
Na (mg)	≤ 357		≤ 357		424	✗

DRV, dietary reference values; kJ, kilojoule; Kcal, kilocalorie; g, gram; N/A, not applicable; mg, milligram; µg, microgram.

* 30 % of the average estimated average requirement (EAR) for energy for boys and girls aged 4, 5 and 6 years^(32)^.

† 35 % of energy (1793 kJ/429 kcal) from total fat^(36)^.

‡ 10 % of energy (1793 kJ/429 kcal) from saturated fat^(34)^.

§ 50 % of energy (1793 kJ/429 kcal) from carbohydrate^(33)^.

|| 30 % of the daily population recommendation for dietary fibre for children aged 2–5 years^(33)^.

** 5 % of energy (1793 kJ/429 kcal) from free sugars^(33)^.

### Recalculation of the nutrient framework for key stage one pupils

The current food-based standards for lunches served in schools are underpinned by a nutrient framework^(17,19)^. This framework was first calculated for the nutrient-based standards which were legislated in 2008 and used until 2014^(31)^. This legislation also included separate nutrient-based standards for KS1 pupils attending infant schools and were based on DRV in place at the time^(20,31)^. Since then, DRV for energy, fibre, free sugars and saturated fat have been revised^(32–34)^. For the purposes of meaningful comparison with current dietary recommendations, the nutrient framework for KS1 pupils has been recalculated for this paper by the researchers based on current DRV. This revised framework has a reduction in energy, fat, saturated fat and carbohydrate, an increase in dietary fibre in comparison to the published framework and the inclusion of free sugars instead of non-milk extrinsic sugars.

### Statistical analysis

As data for some nutrients were not normally distributed and/or displayed unequal variance, the significance of differences between provision of key nutrients between different meal types was explored using Kruskal–Wallis tests in SPSS^(35)^. A value of *P* < 0·05 was used to describe statistical significance, and Dunn’s pairwise post hoc tests with Bonferroni correction were used to determine whether pairs of meal types were significantly different from each other. Means have been calculated for comparison with the nutrient framework as per previous methodology^(19,20)^, but medians are also presented.

## Results

Ten schools were recruited for the study, of which nine provided menu and recipe information and have been included in the nutrient analysis. Lunches were provided by eight different catering providers (either external catering companies, the local authority catering provider or catering provided by the school).

### Comparison with nutrient framework for lunches provided in early years settings

The mean energy and nutrient content of lunches provided for 3–4-year-old children across all nine schools (*n* 161 lunches) was calculated and compared with the nutrient framework for lunches in EYS^(14)^ (Tables 1 and 2). The mean energy content (1881 kJ/450 kcal) was above the nutrient framework (1542 kJ/369 kcal), and no individual school met the nutrient framework for energy. Lunches in the majority (seven) of schools were above the framework, and in the remaining (two) schools below the framework, with the mean energy content of lunches in individual schools ranging from 1371 kJ (328 kcal) to 2366 kJ (566 kcal). The high energy content of lunches resulted in mean fat (15·5 g) and carbohydrate (60·6 g) contents that were also in excess of the nutrient framework, with lunches provided in only two schools (for fat) and one school (for carbohydrate) in line with the framework. The mean percentage energy from fat (31 %) and carbohydrate (51 %) was broadly in line with DRV^(33,36)^, indicating that although absolute amounts of these nutrients provided was high due to excess energy content, the macronutrient composition of lunches was appropriate for these nutrients. The protein content of lunches provided in all nine schools was above the nutrient framework, with a mean protein content (16·8 g) of more than three times the minimum stated in the nutrient framework (5·1 g) and providing 15 % of the energy content of lunches. Due to frequent provision of cakes and biscuits as a dessert option, and the high portion sizes of these^(27)^, the mean free sugars content of lunches across all schools (10·5 g) was more than double the maximum stated in the nutrient framework (4·9 g). No average lunch in any individual school met the nutrient framework for free sugars, with mean free sugars content in individual schools ranging from 5·6 g to 15·6 g per lunch. Lunches across all schools provided a mean of 9 % energy from free sugars, which is significantly higher than the maximum population recommendation of 5 %^(33)^. The mean Na content of lunches across all schools (424 mg) was also higher than the maximum stated in the nutrient framework (≤ 300 mg), with no schools providing lunches in line with the nutrient framework calculated based on maximum population recommendations for this age group^(37)^. Despite concerns about potentially inadequate intake of micronutrients for children of this age, the mean fibre and micronutrient content of lunches across all schools met the minimum amounts stated in the nutrient framework, with the recommendations for fibre, Fe, Zn, Ca, vitamin A and vitamin C each met in at least seven of the nine schools.

### Comparison by meal option

Mean and median energy and nutrient contents of the different menu options provided across all schools were also analysed and compared with the nutrient framework for lunches provided in EYS to examine the impact of meal option on energy and nutrient content (Table 3). Across the nine schools, forty-five main meals, forty-five vegetarian meals, thirty-nine jacket potato meals and thirty-two sandwich meals (each served with accompaniments and desserts) were collected, weighed and nutritionally analysed. Independent samples Kruskal–Wallis tests revealed that there were significant differences in the amount of food provided between different meal options (χ^2^ (3, *n* 161) = 40·59, *P* < 0·001), with median portion sizes of sandwich meals (229 g) significantly smaller than main meals (296 g; *P* = 0·02), vegetarian meals (290 g; *P* = 0·001) and jacket potato meals (353 g; *P* < 0·001). Despite the difference in meal size, there were no significant differences in the median energy content of the different meal options (χ^2^(3, *n* 161) = 0·05, *P* = 0·997), and all were above the nutrient framework, indicating high energy content for all meal options. There were also no significant differences in the median carbohydrate (χ^2^(3, *n* 161) = 2·05, *P* = 0·562), fat (χ^2^(3, *n* 161) = 4·50, *P* = 0·212), saturated fat (χ^2^ (3, *n* 161) = 4·40, *P* = 0·222) or free sugars (χ^2^ (3, *n* 161) = 0·35, *P* = 0·950) content of the different menu options. There were significant differences in protein content by meal type (χ^2^ (3, *n* 161) = 9·48, *P* = 0·024) with significantly higher median protein content in main meals compared with vegetarian options (*P* = 0·014), but both were considerably above the minimum stated in the nutrient framework. Significant differences were also seen in fibre content (χ^2^ (3, *n* 161) = 42·90, *P* < 0·001) with fibre content of sandwich meals significantly lower than the other meal options (*P* < 0·001–*P* = 0·011). There were some significant differences in micronutrient content of the different meal options (vitamin C χ^2^ (3, *n* 161) = 18·01, *P* < 0·001; Fe χ^2^ (3, *n* 161) = 13·86, *P* = 0·003; Ca χ^2^ (3, *n* 161) = 20·06, *P* < 0·001; Na χ^2^ (3, *n* 161) = 19·78, *P* < 0·001), with sandwiches significantly lower in vitamin C (*P* < 0·001–*P* = 0·005) and Fe (*P* = 0·012–*P* = 0·017), but higher in Ca (*P* < 0·001–*P* = 0·05) compared with some or all other meal options. The sandwich meals were also significantly higher in Na than the other meal options (*P* < 0·001–*P* = 0·003).

### Impact of dessert choice

**Fig. 1:**
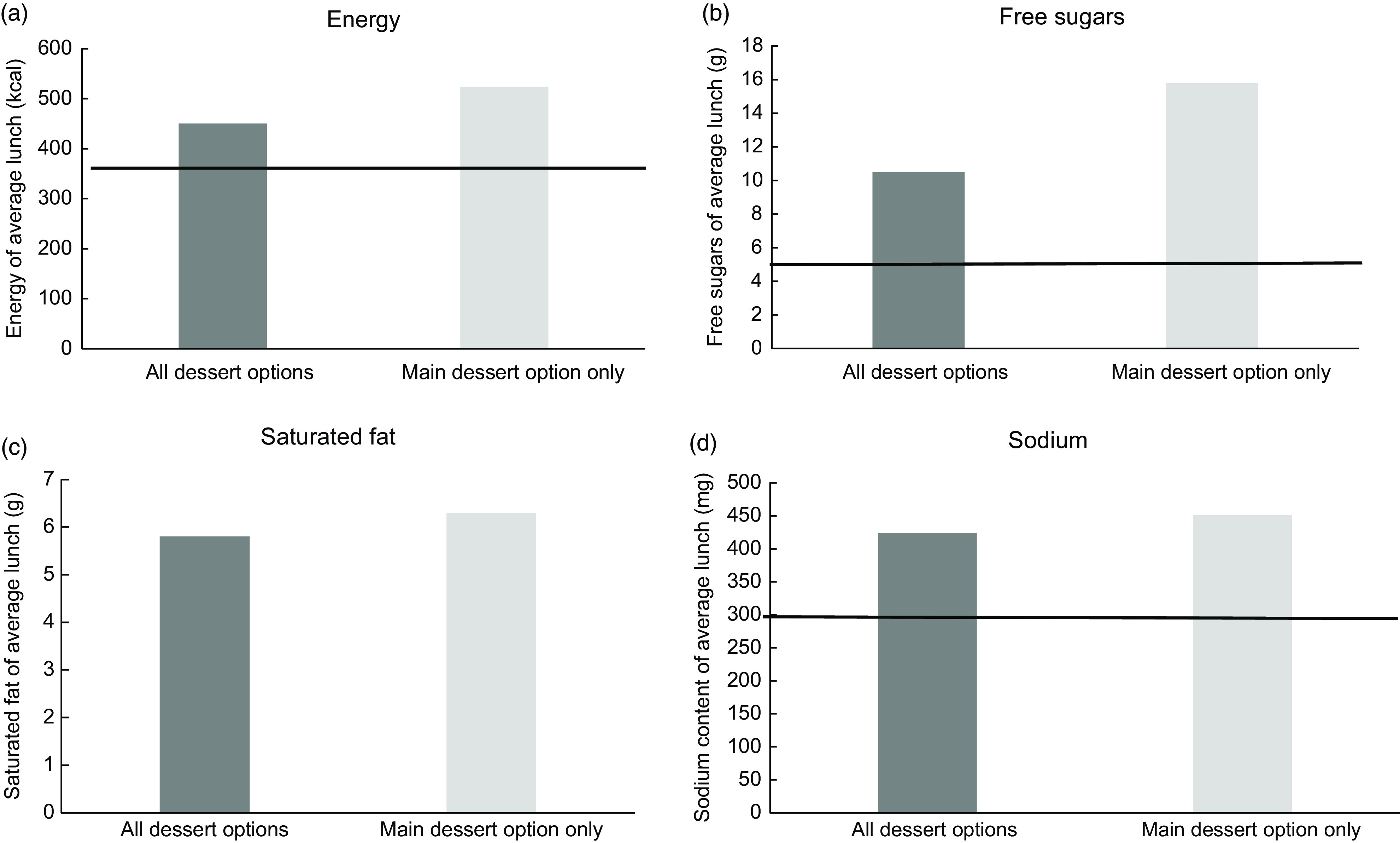
Energy and selected nutrient content of an average main meal (all schools, *n* 9). Comparison of average dessert provision and main dessert option only. Horizontal line represents nutrient framework value.

Figure 1 highlights the impact on selected nutrients in an average lunch across all schools where only the main dessert choice (typically a cake, biscuit or pudding with or without custard) was included in the analysis, compared with all available dessert options. Including only the main dessert led to an increase in energy (from 1881 kJ/450 kcal to 2190 kJ/524 kcal), free sugar (10·5 g to 15·8 g), saturated fat (from 5·8 g to 6·3 g) and Na content (from 424 mg to 451 mg) of the average lunch.

### Comparison with nutrient framework for school lunches provided for key stage one pupils

The mean energy and nutrient content of lunches provided across all schools were also compared with the revised nutrient framework for KS1 pupils underpinning the food-based standards in place for schools. Most of the schools reported that lunches provided to 3–4 year-old children were the same as those provided to KS1 pupils, and schools are likely to plan menus to meet the food-based standards for school lunches which the nutrient framework underpins^(18,19)^ (Table 4). The mean energy content of lunches provided across all schools was just within the revised nutrient framework range for energy (1705–1881 kJ/408–450 kcal). Although average fat content (15·5 g) was in line with the revised nutrient framework (≤ 16·7 g), saturated fat content was too high (5·8 g compared with the framework stating ≤ 4·8 g) indicating that although the DRV does not apply to children under 5 years, the amount provided was in excess of recommendations in place for older children. Levels of carbohydrate (60·6 g) and dietary fibre (6·7 g) in an average lunch met the revised KS1 framework, but the free sugars content of an average lunch was almost twice the maximum amount stated in the framework for KS1 children (≤ 5·7 g). The average lunch provided at least the minimum amounts stated in the nutrient framework for Fe, Ca, vitamin A, vitamin C and folate, but the Zn content did not meet the minimum stated in the nutrient framework (≥ 2·3 mg). The Na content of an average lunch was in excess of the maximum amount stated in the revised nutrient framework for KS1 pupils (≤ 357 mg). These results indicate that if the same lunches are provided to KS1 children as to nursery children as reported by most schools in the study, the amount of energy would be appropriate, saturated fat, free sugars and Na would be in excess of maximum recommendations for this age group, and Zn content may be inadequate to meet requirements.

### Comparison with food-based standards for school lunches and voluntary food and drink guidelines for early years settings

Lunches provided during the week of collection in each school were evaluated against the food-based standards for school lunches^(18)^ and the voluntary food and drink guidelines for EYS relating to lunch^(14)^ (see online supplementary material, Supplemental Table 1). Overall, lunch provision was more compliant with the standards for school lunches than the voluntary guidelines for EYS. Main lunch options provided in all schools met the school food standards for daily provision of a portion of starchy food and variety of starchy food across the week. All schools provided a portion of vegetables or salad as an accompaniment to the main meal, and although all schools met the standard to provide a portion of fruit daily, this was often available as an alternative to the main dessert option rather than as or with the main dessert option, and only four schools provided a fruit-based dessert at least twice a week. All schools provided a portion of protein as part of the main lunch option each day, with meat or poultry provided on at least three days in the week, and seven of the nine schools met the standard for restricting provision of meat and poultry products. The food-based standard most commonly not met was the restriction of starchy foods cooked in fat or oil (such as roast potatoes, potato wedges, garlic bread, Yorkshire pudding and chips) to 2 days a week, with only one of the nine schools meeting this standard. All schools met the standards banning savoury snacks, confectionery and non-permitted drinks. Many of the voluntary guidelines for EYS are aligned to the school food standards, but where these differ, they were commonly not met. The voluntary guidelines for EYS state to limit fried starchy foods to once a week, which was not met in any of the nine schools, neither was the guideline to provide a lunch for all children once a week using pulses nor a meat alternative as the protein. Although cakes not containing confectionery can be provided daily as part of lunch under the school food standards, the voluntary guidelines for EYS state to limit provision of cakes and biscuits to once a week, which was not met in any of the schools.

## Discussion

The aim of the study was to calculate the energy and nutrient content of lunches provided for 3–4-year-old children attending school nurseries, comparing this to the nutrient frameworks underpinning the voluntary guidelines for EYS and the school food standards. The key results of the study show that the average lunch provided for children attending school nurseries had a higher energy, fat, carbohydrate, protein, Na and free sugar content than recommended in the nutrient framework for EYS, although percentage energy from fat and carbohydrate were in line with DRV. The micronutrient and fibre content of the average meal exceeded the values recommended in the framework. When compared with the revised nutrient framework for KS1 pupils, lunches met standards for energy, fat and carbohydrate but provided excess saturated fat, free sugars and Na and insufficient Zn. The energy and nutrient content also varied by meal option, with sandwich meals higher in Ca but also higher in Na and lower in fibre and vitamin C. If only the main dessert option was included, the average lunch was higher in energy, free sugars, saturated fat and Na than when overall dessert provision was included.

Previous research in EYS^(23,24)^ reported lunches to be lower in energy than this study, with means of 404 kcal in a study of twenty nurseries in Liverpool^(23)^ and 306 kcal in the pre-school food survey^(24)^, both of which were below the relevant guidelines used for comparison. Carbohydrate was also lower than this study, with means of 56·2 g and 40·8 g, respectively^(23,24)^. When these previous studies were conducted, lunches were aiming to meet nutrient frameworks based on previous DRV (with higher estimated average requirements for energy for this age group) in place at the time and were conducted in settings only catering for children under 5 years. The schools in this study were also providing lunches for primary-aged children, which may have increased portion sizes in comparison to settings catering purely for nursery-aged children.

The mean energy content of lunches overall (450 kcal) and lunches provided in most individual schools (328–568 kcal) were above the nutrient framework for EYS (369 kcal). This indicates that lunches were providing excess energy in relation to requirements for 3–4-year-old children and were more closely aligned to the requirements of KS1 children (408–450 kcal), further highlighting that menus may be planned with older children, subject to mandatory standards, in mind. The mean protein content of lunches (16·8 g) was more than three times the amount stated in the nutrient framework (5·1 g), which is fairly consistent with results seen in previous studies of KS1 children attending primary schools (18·4 g) and EYS (17·2 g and 12·5 g in the previous study of nurseries in Liverpool and the pre-school food survey, respectively)^(23–25)^. As the protein standard is expressed as a minimum, and protein intakes of this level are reflective of typical intakes for this age group^(4)^, this was not previously highlighted as a concern, and may have positively supported levels of Fe and Zn to meet minimum recommendations set out in the nutrient framework. However, as associations between higher protein intake for children of this age and increased body mass in later childhood are apparent^(5)^, and it has recently been recommended that government consider approaches to reduce excess protein intakes in young children and support children to consume a diet not exceeding energy requirements^(38)^, the high protein content of lunches is a concern, and the high energy and protein content may promote excess intake of these nutrients and risk of weight gain in a group where prevention of obesity is key^(39)^. Mandatory standards for the school lunches provided for nursery children, or clearer guidance for schools catering for both nursery and school children are public health interventions which could help to tackle this issue.

The high free sugars content of children’s diets is also concerning^(33)^, and the free sugars content of lunches in this study was high (10·5 g). Lunches provided more than double the maximum free sugars stated in the nutrient framework for EYS and an average of 9 % of energy came from free sugars, a proportion broadly in line with children’s overall diets (12·1 % of total energy intake)^(4)^. The nutrient framework underpinning the voluntary food and drink guidelines for EYS was revised in 2017 to reflect current dietary recommendations for free sugars introduced in 2015. To help meet this, settings are advised to limit provision of cakes and biscuits to once a week at lunchtime^(14)^, which is consistent with advice to limit consumption of high energy density and discretionary foods for children of this age^(38)^. Conversely, there is no requirement to limit the frequency of provision of cakes and biscuits at lunchtime as part of the mandatory standards for school food, and these were frequently provided as dessert options (with none of the schools limiting provision to once a week) and contained more free sugars than alternative dessert options. School food standards do suggest using fresh or dried fruit to sweeten dishes^(18)^, but the standard to provide fruit-based desserts at least twice a week was only met by four of the nine schools. The nutrient framework underpinning the school food standards is still based on non-milk extrinsic sugars content, but when compared with the revised framework for KS1 pupils, lunches provided almost twice the recommended (5·7 g) maximum for free sugars for this age group. Findings from the nutrient analysis and evaluation against food-based standards and guidance therefore suggest that schools are following school food standards rather than voluntary early years guidance when providing desserts for their nursery pupils, which leads to provision of excess sugar both for the nursery pupils and KS1 pupils and does not support the achievement of dietary recommendations for either age group.

There is some evidence in the UK of inadequate micronutrient intake, including Fe, Zn and vitamin A, amongst children from lower socio-economic status households or from certain ethnic minority groups (Asian or Asian British and Black or Black British)^(38)^, and standards for school food and guidelines for EYS have aimed to compensate for potentially lower intakes of these nutrients outside of school^(19,20)^. Despite this, previous research has indicated that provision of Fe and Zn may be low in lunches provided in EYS. In the Liverpool nursery study, for example, lunches provided 1·8 mg of Fe and 1·9 mg of Zn^(23)^, whilst in the pre-school food survey, figures were 1·5 mg for Fe and 1·4 mg for Zn^(24)^, which for both nutrients is below the guidelines used for comparison. In schools, larger amounts were provided in lunches for KS1 pupils (2·3 mg and 2·1 mg for Fe and Zn, respectively), adequate for Fe (2·1 mg) but below the nutrient-based standard of 2·3 mg for Zn in place at the time^(25)^. In this study, provision of micronutrients overall, and in most individual schools, met the nutrient framework for EYS, and all micronutrients except Zn met the nutrient framework for KS1 pupils. In addition, fibre content, which is typically below recommendations in young children’s diets^(4)^, was also in line with the nutrient framework. However, as the energy content of lunches was approximately 20 % higher than the 1542 kJ/369 kcal stated in the nutrient framework, lunches may need to be more nutrient-dense to ensure continued sufficient provision of micronutrients alongside reduction of energy and protein content.

Reducing the salt content of children’s diets is another key dietary priority, with school food identified as a strategy for enabling this^(40)^. The Na content of lunches was high, with a mean Na content (424 mg), which was above the maximum amounts stated in the nutrient frameworks for EYS (≤ 300 mg) and schools (≤ 357 mg), and with no individual schools meeting the early years framework. This is consistent with findings from previous studies conducted in schools and EYS (with Na contents of 381 mg and 324 mg in the Liverpool nursery study and pre-school food survey, respectively, and 515 mg in school lunches provided for KS1 pupils) and highlights that there is potential for further progress in this area^(4,23–25)^.

The findings also highlight the impact of meal choice on energy and nutrient content. Most schools provided the daily choice between main meals, vegetarian meals, jacket potatoes and sandwich options. Although sandwich meals provided more Ca than main or vegetarian meals (due to the use of cheese as a common filling), they also provided less fibre, vitamin C and Fe and more Na than other meals (due to the bread and common use of ham and cheese as fillings). Meanwhile, the main dessert option provided more energy, saturated fat, free sugars and Na than other dessert options. Limiting the frequency of provision of some items (e.g. cakes and biscuits), following portion size guidance appropriate for the age range being catered for, and ensuring the inclusion of nutrient-dense ingredients as part of sandwich fillings and dessert recipes could all help to reduce excess energy, free sugars, saturated fat and Na content and promote dietary diversity.

Being the first study to assess food provision for children attending EYS within schools is a major strength of this work. Issues faced by caterers are highlighted, such as the challenge of providing nutrient dense options that are not too high in energy and appropriate portion sizes of energy dense foods. Furthermore, this analysis strengthens the case for mandatory standards in EYS and increased support for schools and caterers providing food for multiple age groups.

There are also some limitations that should be considered. Although a range of catering providers were included, and accurate portion size and recipe data collected, schools were purposively sampled and may not be fully representative of all school food provision. Caterers were asked to serve food as if to nursery-aged children but may have made portions larger or smaller to avoid wastage or to use up leftover food. A rigorous and consistent approach to recipe and menu analysis was followed, but some assumptions were made where ingredient information was lacking. Analysis was also based on provision and is not necessarily reflective of consumption. Some choice architecture exists within each menu and children may only consume part of their meal. Children may also have had ad hoc access to an optional salad bar or extra bread, which was not included in the analysis. Finally, many schools provide more portions of some meal types (e.g. main meals) and less of others (e.g. sandwiches) – their ‘provision mix’, which was not reflected in the calculation of the average school meal in each individual school.

### Conclusions

Lunches provided to children attending school nurseries are exceeding the nutrient framework for food provision in early year’s settings and are more in line with the framework for 4–7-year-olds. Saturated fat, free sugars and Na contents of lunches were high when compared with recommendations for both age groups, and the protein content of lunches was significantly higher than amounts stated in both nutrient frameworks. Smaller portions of more nutrient-dense meals may be required, whilst avoiding excessive intakes of protein. Caterers are likely to be more focussed on planning menus to meet mandatory standards in place for school-aged children and updating these standards to reflect current DRV, with a particular focus on reducing free sugars content of lunches (e.g. by reducing the frequency of provision of cakes and biscuits and the portion sizes of these) would support caterers to better meet the nutritional requirements of the nursery and school-aged children they are catering for. In addition, encouraging school caterers to follow the food and drink guidelines for early year’s settings when catering for nursery children would also help ensure appropriate provision for these children. With the proposed extension of free childcare over the coming years^(9)^, it is particularly important to maximise the opportunity to ensure that food provision within EYS supports the development of healthy eating habits and ensures the best start in life for all children.

## Supporting information

Wall and Pearce supplementary material Wall and Pearce supplementary material

## Supplementary material

For supplementary material accompanying this paper visit https://doi.org/10.1017/S1368980023002331
