# Supplementary material for: Energy and nutrient content of school lunches provided for children attending school-based nurseries: a cross-sectional study
Source: Public Health Nutr. 2023 Nov 3;26(12):2641–51. doi: 10.1017/S1368980023002331 (PMC10755416; doi:10.1017/S1368980023002331)
Supplement: Wall and Pearce supplementary material [file S1368980023002331sup001.docx]

Supplementary Table 1 – Evaluation of main meal lunch options served for 3-4 year old children (all schools, n=9) against current food-based standards for school lunches ^(17, 18)^

| Food group | Food-based standard | 1 | 2 | 3 | 4 | 5 | 6 | 7 | 8 | 9 | Total |
| --- | --- | --- | --- | --- | --- | --- | --- | --- | --- | --- | --- |
| Starchy foods | One or more portions of food from this group every day | ✓ | ✓ | ✓ | ✓ | ✓ | ✓ | ✓ | ✓ | ✓ | 9 |
|  | Three or more different starchy foods each week | ✓ | ✓ | ✓ | ✓ | ✓ | ✓ | ✓ | ✓ | ✓ | 9 |
|  | One or more wholegrain varieties of starchy food each week | ✓ | ✓ | 🗶 | 🗶 | 🗶 | ✓ | 🗶 | ✓ | ✓ | 5 |
|  | Starchy food cooked in fat or oil no more than two days each week | 🗶 | 🗶 | 🗶 | 🗶 | 🗶 | 🗶 | ✓ | 🗶 | 🗶 | 1 |
|  | Bread - with no added fat or oil - must be available every day | N/A | N/A | N/A | N/A | N/A | N/A | N/A | N/A | N/A | N/A |
| Fruit and vegetables | One or more portions of vegetables or salad as an accompaniment every day | ✓ | ✓ | ✓ | ✓ | ✓ | ✓ | ✓ | ✓ | ✓ | 9 |
|  | One or more portions of fruit every day | ✓ | ✓ | ✓ | ✓ | ✓ | ✓ | ✓ | ✓ | ✓ | 9 |
|  | A dessert containing at least 50% fruit two or more times each week | ✓ | 🗶 | 🗶 | 🗶 | 🗶 | ✓ | ✓ | 🗶 | ✓ | 4 |
|  | At least three different fruits and three different vegetables each week | ✓ | ✓ | ✓ | ✓ | ✓ | ✓ | ✓ | ✓ | ✓ | 9 |
| Milk and dairy | A portion of food from this group every day | ✓ | ✓ | ✓ | ✓ | ✓ | ✓ | ✓ | ✓ | ✓ | 9 |
|  | Lower fat milk must be available for drinking at least once a day during school hours | N/A | N/A | N/A | N/A | N/A | N/A | N/A | N/A | N/A | N/A |
| Non-dairy protein | A portion of food from this group every day | ✓ | ✓ | ✓ | ✓ | ✓ | ✓ | ✓ | ✓ | ✓ | 9 |
|  | A portion of meat or poultry on three or more days each week | ✓ | ✓ | ✓ | ✓ | ✓ | ✓ | ✓ | ✓ | ✓ | 9 |
|  | Oily fish once or more every three weeks | N/A | N/A | N/A | N/A | ✓ | N/A | N/A | N/A | N/A | N/A |
|  | For vegetarians, a portion of non-dairy protein on three or more days each week | ✓ | ✓ | ✓ | ✓ | ✓ | ✓ | ✓ | ✓ | 🗶 | 8 |
|  | A meat or poultry product…no more than once each week in primary schools | ✓ | ✓ | 🗶 | ✓ | ✓ | 🗶 | ✓ | ✓ | ✓ | 7 |
| Foods high in fat, sugar, salt | No more than two portions of food that have been deep-fried, batter-coated, or breadcrumb-coated, each week | ✓ | ✓ | ✓ | ✓ | ✓ | ✓ | 🗶 | ✓ | ✓ | 8 |
|  | No more than two portions of food which include pastry each week | ✓ | ✓ | ✓ | ✓ | ✓ | ✓ | ✓ | ✓ | ✓ | 9 |
|  | No snacks, except nuts, seeds, vegetables and fruit with no added salt, sugar or fat | ✓ | ✓ | ✓ | ✓ | ✓ | ✓ | ✓ | ✓ | ✓ | 9 |
|  | Savoury crackers or breadsticks can be served at lunch with fruit or vegetables or dairy food | ✓ | ✓ | ✓ | ✓ | ✓ | ✓ | ✓ | ✓ | ✓ | 9 |
|  | No confectionery, chocolate or chocolate-coated products | ✓ | ✓ | ✓ | ✓ | ✓ | ✓ | ✓ | ✓ | ✓ | 9 |
|  | Desserts, cakes and biscuits are allowed at lunchtime. They must not contain any confectionery | ✓ | ✓ | ✓ | ✓ | ✓ | ✓ | ✓ | ✓ | ✓ | 9 |
|  | Salt must not be available to add to food after it has been cooked | N/A | N/A | N/A | N/A | N/A | N/A | N/A | N/A | N/A | N/A |
|  | Any condiments must be limited to sachets or portions of no more than 10 grams or one teaspoonful | N/A | N/A | N/A | N/A | N/A | N/A | N/A | N/A | N/A | N/A |
| Healthier drinks | Free, fresh drinking water at all times | N/A | N/A | N/A | N/A | N/A | N/A | N/A | N/A | N/A | N/A |
|  | Only permitted drinks provided | ✓ | ✓ | ✓ | ✓ | ✓ | ✓ | ✓ | ✓ | ✓ | 9 |

✓food-based standard met; 🗶 food-based standard not met; N/A standard not applicable (e.g. evaluation of standard not possible due to application across the school day, over multiple weeks or related to provision of items outside the core menu).

Supplementary Table 2 – Evaluation of main meal lunch options served for 3-4 year old children (all schools, n=9) against the voluntary food and drink guidelines for early years settings in England relating to lunch provision ^(14)^

| Food group | Food and drink guideline | 1 | 2 | 3 | 4 | 5 | 6 | 7 | 8 | 9 | Total |
| --- | --- | --- | --- | --- | --- | --- | --- | --- | --- | --- | --- |
| Starchy foods | Provide a portion of starchy food as part of each lunch. | ✓ | ✓ | ✓ | ✓ | ✓ | ✓ | ✓ | ✓ | ✓ | 9 |
|  | Provide at least three different starchy foods as part of lunches each week. | ✓ | ✓ | ✓ | ✓ | ✓ | ✓ | ✓ | ✓ | ✓ | 9 |
|  | Provide a variety of wholegrain and white starchy foods each week. | ✓ | ✓ | 🗶 | 🗶 | 🗶 | ✓ | 🗶 | ✓ | ✓ | 5 |
|  | Limit starchy foods which have been fried to once a week at lunch. | 🗶 | 🗶 | 🗶 | 🗶 | 🗶 | 🗶 | 🗶 | 🗶 | 🗶 | 0 |
|  | Limit canned pasta in sauce such as spaghetti hoops. | ✓ | ✓ | ✓ | ✓ | ✓ | ✓ | ✓ | ✓ | ✓ | 9 |
|  | Avoid flavoured dried rice, pasta and noodle products e.g. packets and pots of instant flavoured noodles, pasta and rice. | ✓ | ✓ | ✓ | ✓ | ✓ | ✓ | ✓ | ✓ | ✓ | 9 |
| Fruit and vegetables | Provide a portion of vegetable and/or fruit as part of lunch each day. | ✓ | ✓ | ✓ | ✓ | ✓ | ✓ | ✓ | ✓ | ✓ | 9 |
|  | Provide a variety of vegetables and fruits across the week at lunchtime. | ✓ | ✓ | ✓ | ✓ | ✓ | ✓ | ✓ | ✓ | ✓ | 9 |
| Milk and dairy | It is best practice to provide three portions of milk and dairy foods each day. | N/A | N/A | N/A | N/A | N/A | N/A | N/A | N/A | N/A | N/A |
| Non-dairy protein | Provide a portion of beans, pulses, fish, eggs, meat, or other proteins as part of lunch each day | ✓ | ✓ | ✓ | ✓ | ✓ | ✓ | ✓ | ✓ | ✓ | 9 |
|  | Provide a variety of foods from this group as part of lunches across the week | ✓ | ✓ | ✓ | ✓ | ✓ | ✓ | ✓ | ✓ | ✓ | 9 |
|  | Provide one lunch for all children each week which uses pulses or a meat alternative as the protein source | 🗶 | 🗶 | 🗶 | 🗶 | 🗶 | 🗶 | 🗶 | 🗶 | 🗶 | 0 |
|  | Provide vegetarian children with a variety of protein sources such as pulses, eggs and meat alternatives each week as part of lunch | ✓ | ✓ | 🗶 | ✓ | 🗶 | 🗶 | ✓ | ✓ | 🗶 | 5 |
|  | Provide oily fish at least once every three weeks as part of lunch or tea. | N/A | N/A | N/A | N/A | N/A | N/A | N/A | N/A | N/A | N/A |
|  | Limit bought and homemade meat products to no more than once a week. | ✓ | ✓ | 🗶 | ✓ | ✓ | 🗶 | ✓ | ✓ | ✓ | 7 |
|  | Limit bought and homemade fish products to no more than once a week. | ✓ | ✓ | ✓ | ✓ | ✓ | ✓ | ✓ | ✓ | ✓ | 9 |
|  | Limit bought and homemade products made from meat alternatives to no more than once a week. | 🗶 | 🗶 | 🗶 | ✓ | ✓ | 🗶 | ✓ | ✓ | ✓ | 5 |
| Foods high in fat, sugar, salt | Limit the use of pastry to once a week | ✓ | ✓ | ✓ | ✓ | ✓ | ✓ | ✓ | ✓ | ✓ | 9 |
|  | Avoid salty snacks such as crisps. | ✓ | ✓ | ✓ | ✓ | ✓ | ✓ | ✓ | ✓ | ✓ | 9 |
|  | Limit the use of condiments such as ketchup. | N/A | N/A | N/A | N/A | N/A | N/A | N/A | N/A | N/A | N/A |
| Cakes and desserts | Vary the desserts offered each week and limit provision of cakes and biscuits. | 🗶 | 🗶 | 🗶 | 🗶 | 🗶 | 🗶 | 🗶 | 🗶 | 🗶 | 0 |
| Healthier drinks | Children must have access to drinking water throughout the day and be encouraged to help themselves to water. | N/A | N/A | N/A | N/A | N/A | N/A | N/A | N/A | N/A | N/A |
|  | Provide only fresh tap water/plain milk for children to drink | ✓ | ✓ | ✓ | ✓ | 🗶 | ✓ | ✓ | ✓ | ✓ | 9 |

✓ guideline met; 🗶 guideline not met; N/A guideline not applicable (e.g. evaluation of guideline not possible due to application across the school day, over multiple weeks or related to evaluation of product label).

Within the voluntary food and drink guidelines for early years settings in England, ‘limit’ is defined as to be provided no more than once a week, and ‘avoid’ is defined as not to be provided at all.
